# MORPHEUS, a Webtool for Transcription Factor Binding Analysis Using Position Weight Matrices with Dependency

**DOI:** 10.1371/journal.pone.0135586

**Published:** 2015-08-18

**Authors:** Eugenio Gómez Minguet, Stéphane Segard, Céline Charavay, François Parcy

**Affiliations:** 1 Laboratoire de Physiologie Cellulaire Végétale, Unité Mixte de Recherche 5168, Centre National de la Recherche Scientifique, Commissariat à l’Énergie Atomique, Institut National de la Recherche Agronomique, Université Joseph Fourier Grenoble I, 38054, Grenoble, France; 2 Laboratoire de Biologie à Grande Echelle, CEA/INSERM U1038/UJF—Grenoble 1, IRTSV, F-38054, Grenoble, Cedex 9, France; Univeristy of California Riverside, UNITED STATES

## Abstract

Transcriptional networks are central to any biological process and changes affecting transcription factors or their binding sites in the genome are a key factor driving evolution. As more organisms are being sequenced, tools are needed to easily predict transcription factor binding sites (TFBS) presence and affinity from mere inspection of genomic sequences. Although many TFBS discovery algorithms exist, tools for using the DNA binding models they generate are relatively scarce and their use is limited among the biologist community by the lack of flexible and user-friendly tools. We have developed a suite of web tools (called Morpheus) based on the proven Position Weight Matrices (PWM) formalism that can be used without any programing skills and incorporates some unique features such as the presence of dependencies between nucleotides positions or the possibility to compute the predicted occupancy of a large regulatory region using a biophysical model. To illustrate the possibilities and simplicity of Morpheus tools in functional and evolutionary analysis, we have analysed the regulatory link between LEAFY, a key plant transcription factor involved in flower development, and its direct target gene *APETALA1* during the divergence of Brassicales clade.

## Introduction

The binding of transcription factors (TF) to *cis* elements is a key component of most biological processes. Being able to detect TF binding sites (TFBS) by inspecting genome sequences helps understanding how organisms work and how they evolved. Methods based on Chromatin Immunoprecipitation (ChIP) such as ChIP-Chip [1], ChIP-Seq [2] or ChIP-exo [3] allow the identification of all genomic regions bound by a given TF in one experimental condition and suites as Bedtools [4, 5] offer many tools to manipulate them. To precisely identify the TFBS present in these regions, estimate their affinity, predict binding sites that might be bound in other experimental conditions, or study organism where ChIP experiments are more challenging, TF DNA binding models are extremely useful. There are multiple ways to model TFBS. The most common is the Position Weight Matrix (PWM) that, for each sequence, computes a score directly related to the TF/DNA affinity ([6] for a review). This method, however, assumes that each base of the BS contributes independently to the affinity of the TF for DNA [7] and there is evidence that interdependencies between positions exist [8, 9] and that taking into account dinucleotide dependencies between two adjacent positions already improves predictions [10]. Several alternatives with specific advantages have been proposed using nucleotide subsequences (K-mers) rather than mononucleotide positions [8, 11, 12] or hidden Markov models (HMM) [13]. However, in most cases, PWMs provide simple and reliable estimation of binding affinity [14]. We propose to adapt the convenient PWM model by adding dependency information at specific positions of the matrix. As documented for several TFs [10, 15–17], this will improve the prediction power of some PWM models.

Although several tools such as RSAT [18], PROMO [19], MatInspector [20] or LASAGNA [21] are available to identify overrepresented motifs in a set of sequence and build binding models, none of them allows using PWM with dependencies nor to calculate occupancy of DNA regions using biophysical models [22]. We have developed a new algorithm that uses PWM with any combination of dependent and independent positions. We incorporated it in a user friendly set of tools called MORPHEUS, which offers several specific advantages over existing tools: 1) it is a web tool that does not require any programming skill and can thus be widely used by the biologist community, 2) users can import their own matrices, not only those found in databases, 3) position interdependencies can be included between any positions of the matrix and in combination with independent positions, a possibility currently offered by none of the existing web tools, 4) a global “predicted occupancy” value can be computed for whole DNA regions using a biophysical model [22] that integrates the presence of individual binding sites.

## Results and Discussion

### Morpheus Matrix Format and *m*PWM algorithm

The Morpheus PWM format (*m*PWM) allows the introduction of information on di- or tri-nucleotide dependencies between any indicated positions (not just adjacent ones) within a binding site. Unlike other models that increase model complexity for all positions, *m*PWM conserves the simplicity of a PWM except for interdependent positions. Using *m*PWM, interdependencies are defined as additional 4^(d)^ matrices (d = 2 for dinucleotide dependency, d = 3 for triplets) for any position combination (Example matrix files are provided as S1 Text and S2 Text).

### mPWM Format Conversion Tool

We have provided a tool to generate *m*PWM from an alignment of transcription factor binding sites. Positions with dependency have to be detected using programs such ENOLOGOS [23] and provided as a list of dependent positions for the conversion tool to automatically generate the corresponding 4^(d)^ matrices. Depending on the TF structural features, the possibility is offered to generate symmetric matrices.

### Morpheus tools

The Morpheus suite allows the calculation of relative affinity of TFBS from *m*PWM. Based on the scores of individual binding sites present in a large DNA region, Morpheus also computes the predicted occupancy using a biophysical model as previously described [22, 24, 25]. This possibility, not offered by any other web-tool, is particularly important as individual *cis* elements can vary within a regulatory region even though the occupancy and overall regulation are conserved [16, 26]. The predicted occupancy thus offers a global measure that allows comparing regions independently of the individual binding site variations.

**Fig 1:**
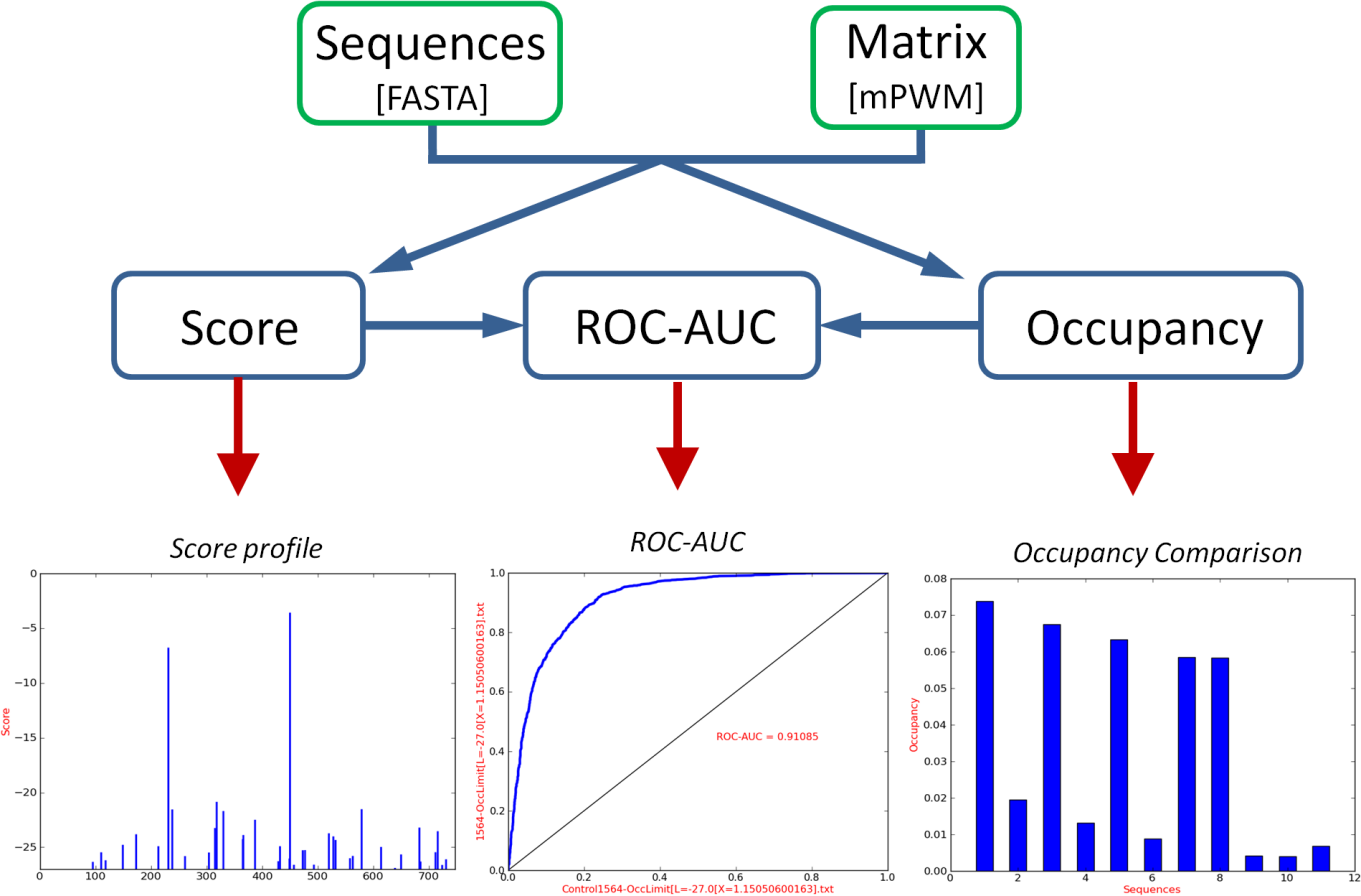
Morpheus flowchart and example of result representation. The tool Score scans DNA regions and computes the scores of TFBS. The bottom left graph shows TFBS locations and scores; such score profile is generated for each sequence submitted. The Occupancy tool computes the TF predicted occupancy of each DNA region taking as input sequence files (in fasta format) and a binding model information (*m*PWM format). Complete results are written in text files and also displayed as graphical outputs for quick results overview. Bottom right panel shows a occupancy comparison between different DNA regions. The bottom central panel illustrates the ROC-AUC curve and value obtained with the ROC-AUC tool.

Morpheus webtool it is composed of three tools:

- Morpheus ‘Score’ tool scans DNA regions and computes the scores of individual TFBS. The user can choose to display only the TFBS of highest score of each region, the TFBS with a score higher than a given threshold score or all TFBS. For an easy graphical representation, this tool also generates score profiles for each sequence as well as an histogram with all scores (Fig 1).

- Morpheus ‘Occupancy’ tool computes the TF predicted occupancy of each DNA region using formalism described above [22, 24] with the option of using only the scores exceeding a given threshold. Occupancy calculation is based on the correlation between predicted score and relative dissociation constant which can be obtained from *in vitro* measurements of relative affinities [27]. If this data is not available a relative occupancy can be calculated using default values for the parameters.

Both score and occupancy options take two files as input: a file with sequences in fasta format and a *m*PWM.

- Morpheus ‘ROC’ allows assessing the quality of a TF binding model by performing a Receiver Operating Characteristics (ROC) analysis [28]. This analysis measures the discriminative power of a TF matrix by comparing a set of bound regions (obtained for example from ChIP-Seq experiments) to a negative control set generated by the user. The comparison uses either the best score TFBS of each sequence or its occupancy and the Area Under the Curve (AUC) value is computed as a measure of the model predictive power.

All three programs display graphic output (Fig 1) for quick results overview and text files with complete results for further analysis by the user.

For illustration of how Morpheus suite works, we present here a set of analyses performed with the LEAFY (LFY) protein, a plant TF with a central role in the evolution and development of flowers [29, 30]. According to *in vitro* affinity measurement a PWM has been proposed for this factor (LFY-Trip) that includes three dependency triplets in a symmetric motif of 19 positions [16], in accordance with the information obtained from the LFY-DNA crystal structures [31, 32].

### LEAFY Binding model evaluation using ROC

**Fig 2:**
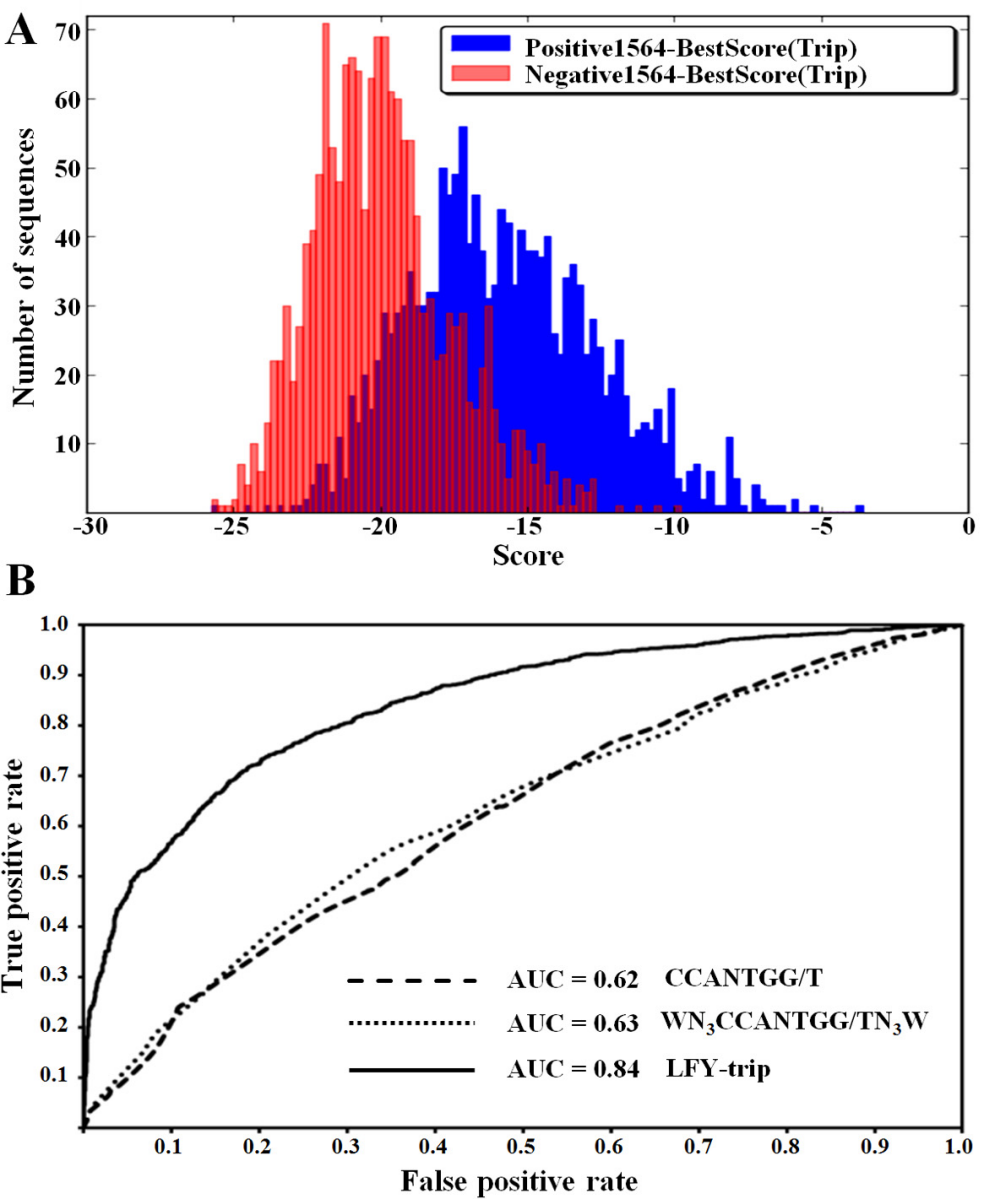
The performance of a TF model can be evaluated by its capability to discriminate between bound and non-bound regions as determined from a ChIP-Seq experiment. The Morpheus ‘ROC’ tools computes the ROC-AUC value as a measure of the model predictive power. **A)** The histogram graphical output displays the distribution of score values for the best binding sites present on each DNA sequence. **B)** ROC data output for three binding models: two consensus motifs and LFY-trip (input data has been generated using the Morpheus ‘Occupancy’ tool). The LFY-trip model largely outperforms the two consensus models.

The availability of ChIP-Seq data allows performing ROC analysis using the described set of bound genomic regions [16] as well as a negative set of non bound regions (see Methods for description of negative set generation) to compare the predictive power of this matrix against the previously described consensus motifs [31, 33, 34]. To do this, Scores or Occupancies are computed for both the positive and negative sets with each binding model (using Score or Occupancy tools) and the result serves as input for the ROC program. A histogram is generated that represents the distribution of scores or occupancy values for each data set (Fig 2A). This tool also generates an image file with the ROC curve and a text file with all the data. In Fig 2B, we use ROC-AUC results to illustrate the increased prediction power of the LFY-Trip PWM as compared to previously used consensus sequences. This tool can be used to compare the various matrices identified by various motifs finding algorithms in order to select the one with the best predictive power. Next, we illustrate how the LFY *m*PWM can be used for functional or evolutionary analysis of a regulatory relationship using the Score and Occupancy tools.

### Prediction of LEAFY binding sites on *APETALA1* promoter

We focused on the link between *LFY* and its direct target *APETALA1* (*AP1*) involved in the development of flowers [29, 30]. The MADS box TF gene *AP1* arose from duplication of the *FRUITFUL* (*FUL*) gene and this event was proposed to be important in the fixation of flower structure in eudicot plants [35]. *AP1* have also experienced a more recent Brassicaceae-specific duplication [36, 37] generating the *CAULIFLOWER* gene. While *AP1* is a direct target of LFY, there is no evidence for direct regulation of *FUL* or *CAL* [16, 38]. We illustrate here how Morpheus can be used to explore *LFY-AP1* link through eudicot plants evolution.

**Fig 3:**
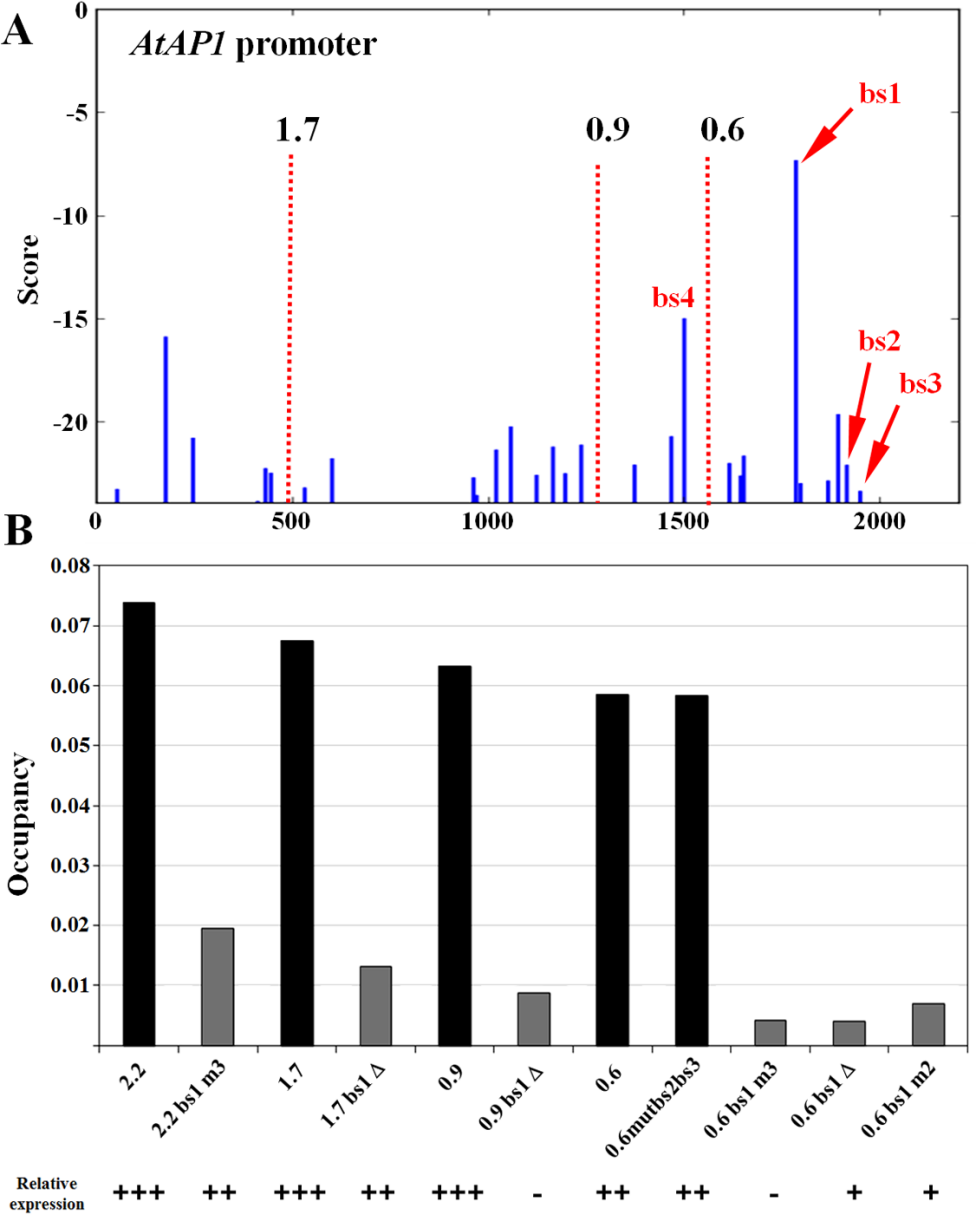
Comparison of LEAFY binding analysis in *A*. *thaliana AP1* promoter using the Morpheus suite with *in vivo* promoter expression study [39]. **A)** Score profile graphic output of the Morpheus ‘Score’ tool (option limit = -25) using 2.2 kb upstream of *AP1* start codon. Red dotted lines show the different promoter sizes and arrows mark the mutated BS, accordingly with promoters set described in [39]. **B)** Predicted occupancy (option All) shows a good correspondence with relative expression of each promoter version as determined experimentally in a published study and summarized: expression levels: +++ (high), ++ (medium), + (low),—(not detectable). The number indicates the size of the promoter (2.2, 1.7, 0.9 or 0.6 kb), m2 and m3 indicate mutations in bs2 and bs3 respectively, ∆1 indicates a deletion of bs1. *In vivo*, mutations of bs2 and bs3 (promoter 0.6 mutbs2bs3) has only a weak effect while elimination of bs1 drastically affects *AP1* expression.

The functional analysis of *AP1* promoter and its regulation by LFY binding has been performed in the model plant *Arabidopsis thaliana*. A few promoter versions have been tested *in vivo* [39] including different promoter lengths (2.2, 1.7, 0.9 and 0.6 kb), and mutations in three candidate LFYBS (bs1, bs2 and bs3) displaying consensus motifs [31, 33, 34]. The score profile of *AP1* promoter generated with Morpheus Score tool (option "limit = -25") illustrates the position of the best LFYBS in *AP1* promoter (Fig 3A). We computed Occupancy values for all promoter versions and compared these values to the *in vivo* activity of the corresponding promoter fragment (Fig 3B). *In vivo*, mutations in bs2 and bs3 had weak effect while mutation in bs1 had a strongest effect, which is in accordance with their computed scores and not with the presence of the consensus sequence. The good correlation between the two types of data illustrates the power of the biophysical model to predict the impact of TFBS changes on gene expression by integrating all possible TFBS present in a regulatory region.

### Transcriptional regulation and evolution

**Fig 4:**
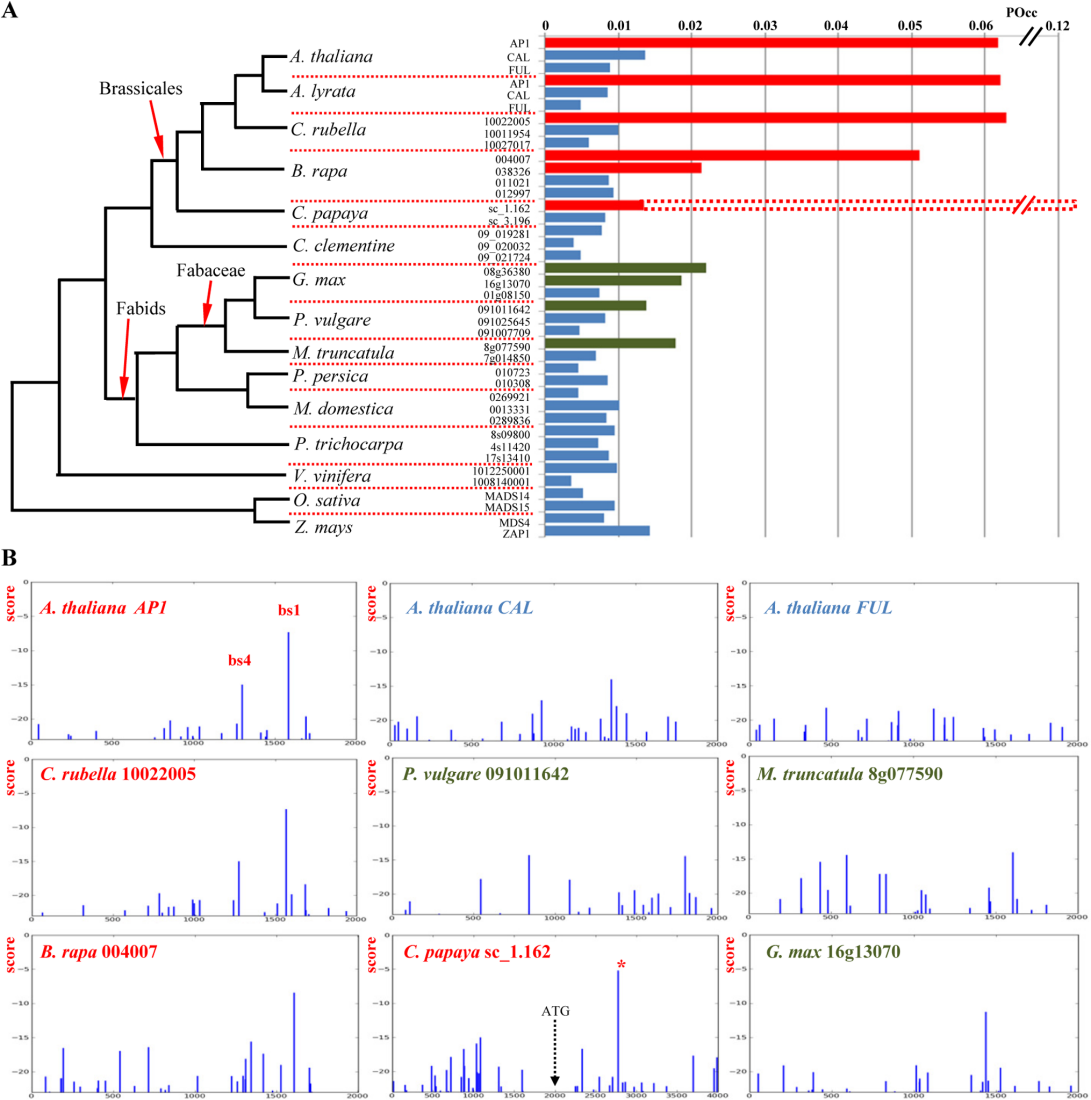
Evolutionary analysis of LFY binding on *AP1* promoters. Genomic sequences were obtained from the Phytozome database and 2 kb promoter upstream the ATG were used. Only well annotated genes were used. **A)** Predicted occupancy (Option limit = -23) for each promoter. Phylogenetic relationships between species are represented. **B)** Score profile (limit = -23) of some representative promoters. The higher occupancy for *AP1* promoters in Brassicales (red) suggests that the regulatory link between LFY and *AP1* in *A*. *thaliana* arose before the divergence of this clade. Interestingly, *C*. *papaya* with low occupancy in *AP1* promoter has a candidate BS of very good score downstream the start codon likely to be responsible for a regulation by LFY. In Fabids, the low occupancy values suggest that LFY does not regulate *AP1*, though some promoters have intermediate occupancy values (green) what will need further analysis.

Next, we use the Morpheus tools to study the evolution of the link between LFY and genes of the *FUL* clade (*AP1*, *CAL* and *FUL*). Scanning of 2 kb of promoter sequences in various species illustrates well the diversity of LFYBS landscapes (Fig 4B). As it is difficult to draw clear conclusions directly from these TFBS profiles, we computed the Occupancy (Occ) for these different promoters (Fig 4A). We found a higher occupancy of the *AP1* ortholog promoters as compared to those of *CAL* and *FUL* in the Brassicales clade, a result in good accordance with experimental data available in Arabidopsis [16, 33, 38, 40]. This analysis suggests that the link between LFY and *AP1* originated before the divergence of *B*. *rapa*.

Interestingly, the promoter of the *AP1* gene from the Brassicale *Carica papaya* displays a low occupancy though evidence suggests a regulation by LFY. We wondered whether this reflected an absence of regulatory link between LFY and *AP1* in this species or whether the LFY binding sites could be located outside of the promoter. We thus scanned the region downstream of the start codon and we found a predicted BS with a very good score that could be responsible for the *AP1* regulation by LFY in this species despite the absence of high affinity LFYBS in the promoter. None of the other species with low promoter occupancy displayed this 3’ binding site (data not shown). This data supports the hypothesis that LFY-*AP1* link originated before Brassicales divergence. The low occupancy values for *AP1* promoters in Fabids suggest LFY does not regulate *AP1* in these species. However, because there are intermediate Occupancy values in the Fabaceae clade, a more detailed experimental characterization would be required in these species to assay the possible existence of a regulatory relationship between LFY and *AP1*.

These analyses illustrate how genomic sequences can be analysed with the Morpheus tool to generate hypotheses regarding gene regulation and regulatory network evolution. More examples can be found in three additional studies [16, 41, 42] that used Morpheus while under development. As more TF binding models become available, such tools will become increasingly important to exploit the genomic data, answer evolutionary questions and bringing up new working hypotheses.

## Conclusions

Morpheus web allows a user-friendly suite of tools for the calculation of TFBS relative affinity on DNA sequences. It incorporates unique features such as dependency between specific positions, occupancy calculation and ROC-AUC estimation that do not exist in any currently available webtool. We have illustrated how it can be used to infer hypothesis about TFBS functional significance or about evolution of regulatory links. Experienced users can download Morpheus scripts code for specific purpose, however no programming skills are needed to use Morpheus web-tools. With all its unique characteristics and with the possibility of using any own-modified *m*PWM, we believe Morpheus should have strong acceptance among biologists. Morpheus web-tools, complete user guide and downloading versions are available at Morpheus website: http://biodev.cea.fr/morpheus/.

## Methods

### Morpheus

All scripts for Morpheus tools are written in Python programming language (ver 2.6.7). The graphic output requires two modules: Numpy (http://numpy.scipy.org/) and Matplotlib (http://matplotlib.sourceforge.net/). Morpheus tools are available in the Morpheus web (http://biodev.cea.fr/morpheus/), as well as downloading versions with or without graphic output, user guide and complete descriptions. The web is hosted and maintained by the GIPSI team (CEA Saclay).

When the score matrix is not directly provided, Morpheus computes it based on ‘Count’ or ‘Frequency’ matrices using *W*_*n*,*i*_ = *Ln(f*_*n*,*i*_ */f*_*max*,*i*_ *)* where *W*_*n*,*i*_ is the weight at position *i* for nucleotide *n*, *f*_*n*,*i*_ is the frequency of nucleotide *n* at position *i* and *f*_*max*,*i*_ is the maximal frequency observed at position *i* [43]. Each 4^(d)^ dependency matrix is preceded by a line indicating the positions involved (S2 Text). For score calculation the *m*PWM algorithm first get the value for each independent position from the independent matrix and then for all the dependency combinations from the 4^(d)^ matrices. If *in vitro* affinity data is available to correlate score with relative dissociation constant, the correlation values can be indicated in *m*PWM file or, if they are not indicated, the program will use default parameters (corresponding to a line curve with scope equal to one). From matrix file, *m*PWM algorithm first identifies the list of independent positions (*i*) and the list of dependent positions groups (*j*; each one associated with a 4^(d)^ matrix), then the score of each DNA sequence is calculated as:

$$Sequence_{score}=\sum_{p\in i}score_{p_{nt}}+\sum_{q\in j}score_{q_{dep}}$$

where $score_{p_{nt}}$is the score in the position *p* for the nucleotide *nt* (A,C,G or T) in the independent matrix and $score_{q_{dep}}$is the score in the 4^(d)^ matrix of group *q* for the sequence combination *dep* (dinucleotide or triplet). Example of matrix files in Morpheus format with or without dependencies are provided as S1 Text and S2 Text, respectively.

### Occupancy calculation (default parameters)

Occupancy calculation is based on the relation between predicted score and relative dissociation constant [16, 24], score = -a * ln(Kd) + b. A and b values can be provided in the *m*PWM file when they have been determined. If not, Morpheus will use default parameters (a = 1.0 and b = 0.0). Occupancy calculation formalism also requires the TF concentration [X], which can optionally be indicated if available. Since this value is rarely available, as default, Morpheus uses [X] = e ^(b/a)^ corresponding to a TF concentration at which the best possible binding site (maximal score) is bound with a probability of 0.5.

### Sequences sets for ROC-AUC calculation

Positive bound sequences was taken from [16] (S3 Text). To generate a negative set of sequences for ROC-AUC analysis, we have randomly selected in the *A*. *thaliana* genome a set of sequences that do not overlap with the positive set and with the same size distribution (S4 Text).

### *FUL* clade genomic sequences

Genomic sequences were obtained from Phytozome database [44] by Blast search using the protein sequence of *AtAP1* (At1g69120), *AtCAL* (At1g26310) and *AtFUL* (At5g60910). Hits without transcripts information or with incomplete gene prediction were discarded. A region of 2 kb upstream the ATG were used for relative binding score calculation. All sequences used in this study can be found in S5 Text.

## Supporting Information

S1 Text Example of mPWM format without dependency. (TXT)

S2 Text Example of mPWM format with dependency. (TXT)

S3 Text Positive Sequences Set. (TXT)

S4 Text Negative Sequences Set. (TXT)

S5 Text FUL clade genomic sequences. (TXT)
